# Altered White Adipose Tissue Protein Profile in C57BL/6J Mice Displaying Delipidative, Inflammatory, and Browning Characteristics after Bitter Melon Seed Oil Treatment

**DOI:** 10.1371/journal.pone.0072917

**Published:** 2013-09-06

**Authors:** Cheng-Hsien Hsieh, Gou-Chun Chen, Pei-Hsuan Chen, Ting-Feng Wu, Pei-Min Chao

**Affiliations:** 1 Institute of Nutrition, China Medical University, Taichung, Taiwan; 2 Department of Biotechnology, Southern Taiwan University of Science and Technology, Tainan, Taiwan; University of Santiago de Compostela School of Medicine - CIMUS, Spain

## Abstract

**Objective:**

We have previously shown that bitter melon seed oil (BMSO), which is rich in *cis*-9, *trans*-11, *trans*-13 conjugated linolenic acid, is more potent than soybean oil in attenuating body fat deposition in high-fat diet-induced obese C57BL/6J mice. The aim of this study was to obtain a comprehensive insight into how white adipose tissue (WAT) is affected by BMSO administration and to explore the underlying mechanisms of the anti-adiposity effect of BMSO.

**Methods and Results:**

A proteomic approach was used to identify proteins differentially expressed in the WAT of mice fed diets with or without BMSO for 11 wks. The WAT was also analyzed histologically for morphological changes. Two-dimensional gel electrophoresis (pH 4–7) revealed 32 spots showing a statistically significant difference (*P*<0.05) in intensity in BMSO-treated mice and 30 of these were shown to code for 23 proteins (15 increased and 8 decreased expression; >2-fold change). Combined with histological evidence of macrophage infiltration and brown adipocyte recruitment, the proteomic and immunoblotting data showed that the WAT in mice subjected to long-term high dose BMSO administration was characterized by reduced caveolae formation, increased ROS insult, tissue remodeling/repair, mitochondria uncoupling, and stabilization of the actin cytoskeleton, this last change being putatively related to an increased inflammatory response.

**Conclusion:**

The anti-adiposity effect of BMSO is associated with WAT delipidation, inflammation, and browning. Some novel proteins participating in these processes were identified. In addition, the BMSO-mediated WAT browning may account for the increased inflammation without causing adverse metabolic effects.

## Introduction

Obesity has become a worldwide epidemic and is causally linked with many metabolic disorders and cancers [1]. Besides eating less and exercising more, effective and safe agents that can be used as adjuncts to decrease body fat deposition are being sought. Many reports have shown that conjugated fatty acids, i.e. polyunsaturated fatty acids with a conjugated diene or triene, are less adipogenic than non-conjugated fatty acids and might be helpful in weight reduction [2]–[6]. Of these, conjugated linoleic acid (CLA), with an anti-adipogenic effect mainly attributed to the *trans*-10, *cis*-12 (*t*10, *c*12)- isomer, has been the most studied functionally and mechanistically. However, in mice, *t*10, *c*12-CLA alone or a *t*10, *c*12-CLA and *c*9, *t*11-CLA mixture elicits lipodystrophy with comorbidity of insulin resistance and hepatic steatosis, which raises concerns for human application [2], [3]. In addition, commercial CLA, a 1∶1 mixture of *c*9, *t*11-CLA and *t*10, *c*12-CLA, is chemically synthesized. We therefore examined the effects of the natural product, *cis*-9, *trans*-11, *trans*-13-conjugated linolenic acid (*c*9,*t*11,*t*13*-*CLN), otherwise known as α-eleostearic acid, which is abundantly present in bitter melon.

In addition to its well-recognized hypoglycemic effects, bitter melon (*Mormordica charantia)*, a vegetable commonly eaten in Asia, has recently been shown to have anti-obesity effects in rodents [7]–[9]. Using the 3T3-L1 preadipocyte cell line, we have previously shown that *c*9,*t*11,*t*13*-*CLN is far less potent than its non-conjugated counterpart, linolenic acid, or other common unsaturated C18 fatty acids in stimulating adipocyte differentiation, which can be partly attributed to its apoptotic effect on proliferating and differentiating preadipocytes [10]. Using bitter melon seed oil (BMSO) as a natural source of *c*9,*t*11,*t*13*-*CLN (50% of the total fatty acids), we carried out an animal study to show that BMSO is more potent than soybean oil (SBO) in attenuating high-fat diet-induced body fat deposition [11]. Although long-term high-dose BMSO administration resulted in increased numbers of crown-like structures and apoptotic nuclei in the white adipose tissue (WAT), implying macrophage infiltration and apoptosis are involved in the delipidative effect of BMSO, no adverse effects on insulin sensitivity and liver/serum lipids were observed in BMSO-treated mice [11].

Investigation of the signaling pathways associated with energy homeostasis showed that BMSO increases cAMP-activated protein kinase (PKA) and leptin signaling in the WAT [11]. It is known that activation of PKA by sympathetic innervation induces not only lipolysis via phosphorylation of hormone sensitive lipase and perilipin, but also the appearance of brown-like adipocytes in the WAT [12], [13]. Increased leptin signaling in the WAT has been shown to transform the white adipocytes into fat-burning cells [14]. These results raise the possibility that a white-to-brown fat phenotypic switch might occur in the WAT in BMSO-fed mice.

White and brown adipocytes are two distinct subtypes present in adipose tissues; the former are highly adapted to store excess energy and the latter, by oxidizing chemical energy to produce heat, contribute to non-shivering thermogenesis. The browning capacity of WAT in different depots and strains is highly variable and this is postulated to be, in part, attributable to the abundance of the brown-in-white (brite) precursor population. These cells are a subset that look like white adipocytes in the basal state, but with high potential to convert into a thermogenic brite phenotype when stimulated by β-adrenergic or PPARγ agonists [15]–[17]. Although the stimulated brite/beige cells express UCP1 and display a multilocular morphology characteristic of brown adipocytes, they are regarded as different from the “classical” brown adipocytes in interscapular brown adipose tissue (BAT), with a distinct lineage and specific molecular signatures [16]. Many studies in rodents have shown that the increased formation of brite cells as a result of genetic or pharmacological manipulation results in anti-obesity and anti-diabetes phenotypes [17]–[19].

In the present study aimed at obtaining a comprehensive insight into how WAT is affected by BMSO administration and to explore the underlying mechanisms of the anti-adiposity effect of BMSO, a proteomic approach was used to identify proteins differentially expressed in the WAT of mice fed a SBO-based high-fat diets without (control) or with BMSO. The proteomics data combined with the histological and immunoblotting results demonstrated that the WAT in BMSO-fed mice was characterized by delipidation, inflammation, and browning, and some novel proteins participating in these processes were identified.

## Materials and Methods

### Ethics Statement

All experimental procedures were in compliance with all applicable principles set forth in the National Institutes of Health Guide for the Care and Use of Laboratory Animals (Publication No. 85-23, revised 1996). The protocols for animal care and handling were approved by the Institutional Animal Care and Use Committee of the China Medical University (protocol No 97-12-N).

### Animals and Diets

This batch of animals is the same as that used in our previous study on the anti-adiposity effect of BMSO [11]. Male C57BL/6JNarl mice at 9 wk of age were divided into four groups and fed SBO-based high-fat diets containing different percentages of BMSO [30% SBO (HS), 25% SBO+5% BMSO (LBM), 20% SBO+10% BMSO (MBM), or 15% SBO+15% BMSO (HBM)] for 11 wk. The diets for the HS, LBM, MBM, and HBM groups therefore contained 0, 2.5, 5, or 7.5% *c*9,*t*11,*t*13*-*CLN, respectively. All four diets were designed to have 54% of the energy from fats, 26% from carbohydrates, and 20% from proteins. For proteomic and histological studies, retroperitoneal fat from the HS (control) and HBM groups was used. To validate the differential expression of some proteins, retroperitoneal fat from all four groups was examined by immunoblotting.

### Preparation of WAT Protein Lysates

WAT was ground under liquid nitrogen in mortar and the powder homogenized in lysis buffer [7 mol/L urea, 2 mol/L thiourea, 100 mmol/L dithiothreitol, 4% (v/v) 3-[(3-Cholamidopropyl)dimethylammonio]-1- propanesulfonate (CHAPS), 40 mmol/L tris-base (pH 10), 1 mmol/L phenylmethylsulfonyl fluoride, and 1 Complete Mini protease inhibitor cocktail tablet (Roche Diagnostics, Indianapolis, IN, USA) per liter] and centrifuged at 14,000×g for 20 min, then the supernatant was centrifuged at 349,000×g. After centrifugation, the supernatant was cleaned with a 2D clean-up kit (Amersham-Pharmacia Biotech, Piscataway, NJ, USA), and the protein pellet was dissolved in rehydration buffer (7 mol/L urea, 2 mol/L thiourea, 4% CHAPS, 2% dithiothreitol, 0.5% IPG buffer, and a trace amount of bromophenol blue) and stored at −80°C until use. The protein concentration was determined using the *Bio-Rad* protein assay kit (Biorad, Hercules, CA, USA).

### Two-dimensional Gel Electrophoresis (2-DE) and Comparison

The immobilized pH gradient dry strips (Amersham Pharmacia Biotech, pH 4–7, 18 cm) were rehydrated for 16 h with 320 µL of rehydration buffer containing 100 µg protein lysates. A *2*D plus one silver staining kit (Amersham-Pharmacia Biotech Inc.) was utilized to detect proteins, with slight modification as described elsewhere [20]. Images of the 2-DE gels were captured using a BioRad GS800 densitometer. To search for proteins showing differential expression, 15 pairs of well-focused proteome maps were compared using PDQuest 8.0.1 (BioRad) software. Spots with a differential intensity of ≥ 2-fold and present in at least 10 of the 15 gel pairs were regarded as potential targets.

### Protein Identification

The in-gel digestion and mass spectrometric analysis were performed as described previously [20]. Briefly, the protein digest was separated in a LTQ-Orbitrap hybrid tandem mass spectrometer (ThermoFisher, San Jose, CA, USA) in-line coupled with an Agilent 1200 nanoflow HPLC system equipped with an LC Packing C18 PepMap 100 (length: 5 mm; internal diameter: 300 µm; bead size: 5 µm) as the trap column and an Agilent ZORBAX XDB-C18 (length: 50 mm; internal diameter: 75 µm; bead size: 3.5 µm) as the separating column. TurboSequest program (ver. 27, rev. 11) was then used to search for the best matched peptides from a mouse protein database. While only the tryptic peptides with ≦ 2 missed cuts were considered, the mass ranges during the database search were 1 and 3.5 *m/z* for fragment and precursor ions respectively. The protein identities were verified only when there were at least two peptides matched. If more than one protein was identified, the search result with the highest Xcore (i.e. ≧ 2.0 for doubly charged peptides and ≧ 3.0 for triply charged ones) and with minimal differences between experimental and theoretical masses was regarded.

### Immunoblotting

WAT was homogenized in RIPA buffer containing 1% protease inhibitor cocktail and samples containing 60 µg of protein subjected to electrophoresis on 10% SDS gels, transferred to a PVDF-Plus membrane (NEN Life Science, Boston, MA, USA), and immunoblotted. The primary antibodies, used at a dilution of 1∶1000 in PBS, were mouse antibodies against Xenopus β-actin (Novus Biologicals, Littleton, CO, USA) or bovine cellular retinol binding protein 1 (CRBP1) (Santa Cruz Biotechnology, Dallas, Texas, USA) or rabbit antibodies against human swiprosin 1 (Sigma-Aldrich, St Louis, MO, USA), human extracellular superoxide dismutase 3 (EC-SOD3), human uncoupling protein 1 (UCP1), human γ-synuclein, or human cathepsin D (all from Abcam, Cambridge, UK), human caveolin 1 (CAV1), horse cytochrome c (both from Santa Cruz), and human light chain 3 (LC3) (from Cell Signaling, Danvers, MA, USA). HRP-labeled donkey anti-rabbit IgG antibodies or rabbit anti-mouse IgG antibodies (Amersham International) at a dilution of 1∶5000 in PBS were used as secondary antibody. Bound antibodies were detected using an enhanced chemiluminescence Western blotting kit (Amersham International) and the images quantified by densitometric analysis using a Multimage Light Cabinet (Alpha Innotech Corporation, San Leandro, CA, USA).

### Immunohistochemical Analysis

Retroperitoneal fat fixed in 10% formalin was dehydrated through a graded ethanol series, embedded in paraffin, and cut into 5 µm sections. After deparaffinization and rehydration, the sections were incubated with 0.5% Triton X-100, then blocked using 5% goat serum in PBS. The primary antibodies, used at a dilution of 1∶100 in PBS, were rabbit antibodies against mouse CD68 (Abbiotec, San Diego, CA, USA) or human UCP1 (Abcam), while the secondary antibodies were Alexa Fluor 594-labeled goat anti-rabbit IgG antibodies (Invitrogen, Carlsbad, CA, USA) for CD68 or biotinylated goat anti-rabbit IgG antibodies (Dako, Carpinteria, CA, USA) for UCP1, both at a dilution of 1∶250 in PBS. Images of CD68 staining were acquired using a fluoromicroscope equipped with a SPOT RT color-2000 digital camera (Diagnostic Instruments, Sterling Heights, MI, USA), while sections for UCP1 staining were processed using a Dako kit (Dako REALTM envision TM detection system) according to the manufacturer’s instructions and examined on a BX60 microscope (Olympus, Melville, NY, USA).

### RNA Isolation and mRNA Detection

Total RNA was extracted from retroperitoneal fat using TRIZOL reagent according to the manufacturer’s instructions (Invitrogen). Total RNA (1 µg) was reverse-transcribed into first-strand cDNA using 200 units of MMLV-RT in a total volume of 20 µL. For real-time PCR, an SYBR system with self-designed primers was used (Table S1 **)**. Amplification using forty cycles of two steps (95°C for 15 s and 60°C for 1 min) was performed on an ABI Prism 7900HT sequence detection system.

### Fatty Acid Analysis

The lipids extracted from the epididymal fat were subjected to transesterification by the sodium methoxide method [21], and the fatty acid methyl esters generated were dissolved in hexane for fatty acid analysis in a Hewlett-Packard 5890 GC using flame ionization detection on a DB-1 fused silica capillary column (60 m×0.25 mm×0.1 um, Agilent, Inc, Palo Alto, CA, USA) with nitrogen as carrier gas (1.5 mL/min). The fatty acid peaks were identified by comparison of the retention times with those of authentic standards.

### Statistical Analysis

Data are expressed as the mean ± SE. Significant differences between two groups were analyzed by Student’s *t* test. If the variances were not homogeneous, the data were transformed to log values for the statistical analysis. The General Linear Model of the SAS package was used for statistical analyses, and differences were considered significant at *P*<0.05.

## Results

### Histochemical Study

**Figure 1:**
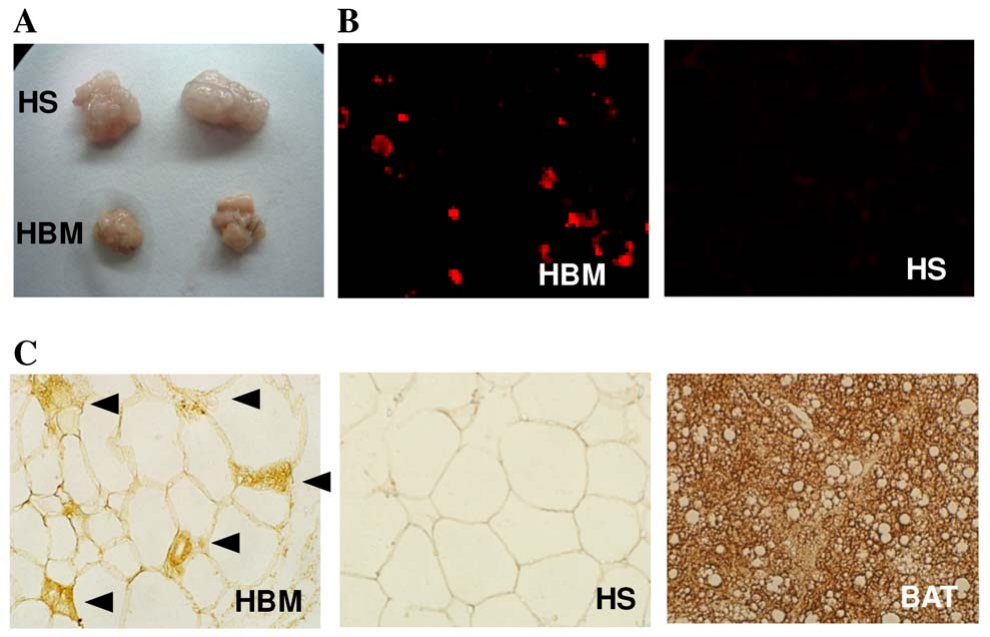
Chronic high-dose BMSO administration results in macrophage infiltration and brown-like adipocyte recruitment in the WAT. Representative picture of retroperitoneal fat pads (**A**) and immunohistochemical results for staining for CD68 (**B**, red) or UCP1 (**C**, brown) in the retroperitoneal fat of mice fed the SBO-based high-fat diet alone (HS, control) or containing high dose BMSO (HBM) for 11 weeks. In (C), brown fat-like cells (indicated by arrowheads) containing multilocular lipid droplets and expressing UCP1 are present in the WAT in the HBM group. A representative image of a UCP-1-stained BAT section is also shown (right panel).

With energy intake controlled to be equal, the body fat (retroperitoneal, epididymal, and inguinal fat) in the LBM, MBM, and HBM groups was significantly reduced, respectively, by 38, 42, and 73% compared to the HS control, and this dose-dependent reduction in adiposity was supported by changes in the serum leptin concentration [11]. Figure 1A shows that the retroperitoneal fat pads dissected from the HBM group were not only smaller, but also darker, than those obtained from the HS control. Figure 1B shows the results of immunostaining of the retroperitoneal fat for CD68, confirming that macrophage infiltration occurred in the WAT of mice in the HBM group, but not the HS group. As shown in Figure 1C, in contrast to the multilocular morphology of the brown adipocytes in the BAT (right panel), adipocytes in the WAT (left and center panels) had a unilocular morphology. However, brown fat-like cells with a multilocular morphology and expressing UCP1 were found to be interspersed in the WAT in the HBM group (left panel), but not the HS group (center panel).

### Proteomic Analysis

**Figure 2:**
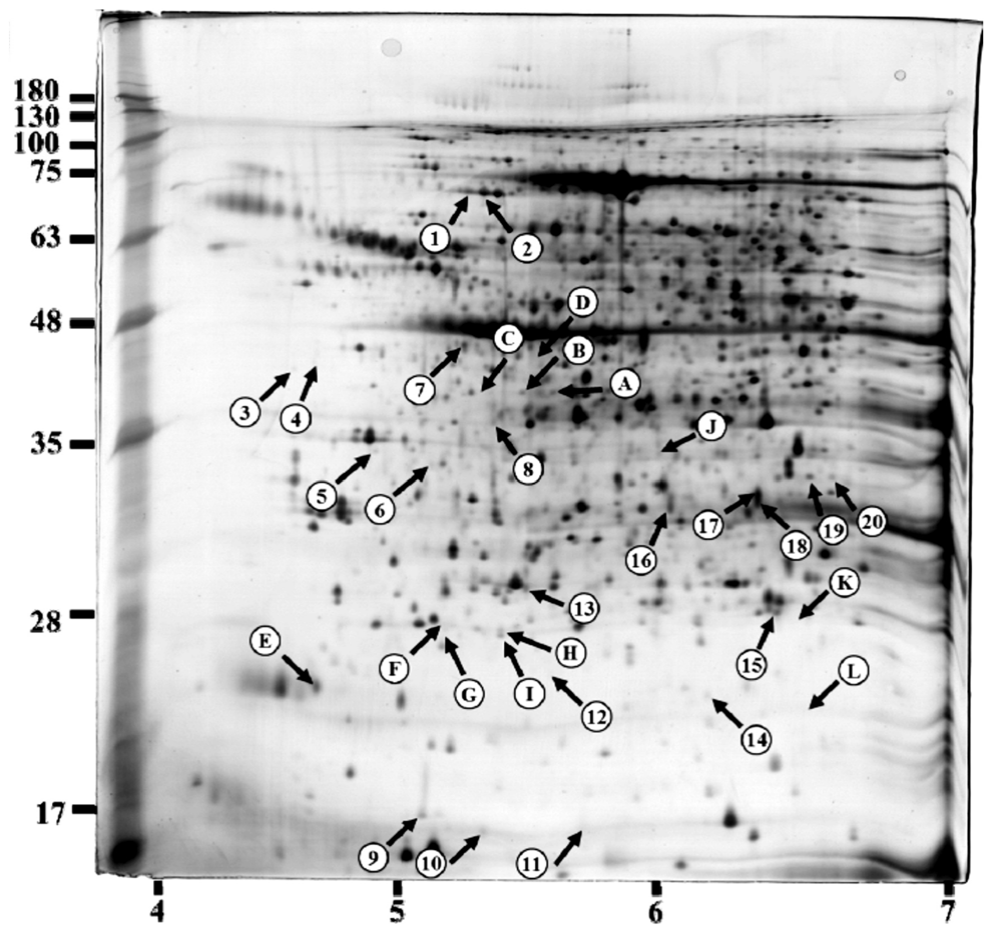
Silver-stained 2-DE polyacrylamide gel of a WAT sample from a HS mouse. Protein lysates were prepared from retroperitoneal fat as described in the Materials and Methods, and 100 µg of protein loaded on a linear pH gradient strip, followed by vertical separation on a 12.5% SDS polyacrylamide gel. The numbers at the bottom of the gel indicate the pH range and those on the left indicate the approximate molecular mass (kD) determined using Bio-Rad protein markers. The 32 spots with an altered intensity in HBM mice compared to HS mice are indicated by arrows. Twenty were more intense (spots 1–20) and 12 less intense (spots A-L) in the HBM group.

In order to investigate the mechanism underlying the BMSO-evoked morphological changes in the WAT, proteome maps from HBM-treated and HS control mice were compared using 2-DE gel-based proteomics to identify differentially expressed proteins. Figure 2 shows a representative proteome map of the WAT from an HS control. The proteins were well separated in a 18 cm gel with a pH range of 4–7. Imaging analysis showed that, on average, the gel resolved 1154±227 and 1180±240 protein spots in the HS and HBM group, respectively. Although the spot profiles on the control and HBM gels were similar, 32 spots showed a significantly different (*P*<0.05) intensity in the 2 groups (Figure 2), 20 being more intense (denoted by numbers) and 12 less intense (denoted by letters) in the WAT from the HBM mice. Areas in the gel containing each of these spots are shown amplified in Figure S1. Thirty spots were identified as originating from 23 proteins, 15 of which were upregulated and 8 downregulated by a factor of ≥ 2-fold in the HBM mice (Table 1). Table S2 shows the theoretical and experimental isoelectric point and molecular weight of proteins identified.

**Table 1:** Proteins differentially expressed in the WAT of HBM mice compared to HS mice identified by MS^1^.

SN	NCBI accessionnumber	Protein	Function	Fold difference(HBM/HS)
**1.**	148703887	Lymphocyte cytosolic protein I/L-plastin	Cytoskeleton stabilization	4.37
**2.**	148703887	Lymphocyte cytosolic protein I/L-plastin		4.44
**3.**	–	Nonidentified	–	3.37
**4.**	11875203	Tropomyosin beta chain	Cytoskeleton stabilization	3.16
**5.**	1098603	Annexin V	?	8.18
**6.**	116138229	Swiprosin 1/Efhd2	F-actin binding protein	8.20
**7.**	123298589	Actin, γ, cytoplasmic 1	Cytoskeleton stabilization	4.43
**8.**	12842843	Inorganic pyrophosphatase	Lipid metabolism	3.92
**9.**	12841904	Coactosin-like protein (CLP)	F-actin binding protein	7.07
**10.**	12847899	Cellular retinol binding protein 1 (CRBP1)	Retinol binding protein	5.68
**11.**	–	Nonidentified	–	20.07
**12.**	123298604	Mitochondrial ribosomal protein L12 (MRPL12)	Mitochondrial protein	3.63
**13.**	160333304	Apolipoprotein A-I preproprotein	Lipoprotein metabolism	4.61
**14.**	148685182	Demethyl-Q7, isoform CRA_a	Mitochondrial protein	7.93
**15.**	123230136	Peroxiredoxin 1	Antioxidation	9.14
**16.**	148705707	Extracellular superoxide dismutase 3 (EC-SOD3)	Antioxidation	6.52
**17.**	115718	Cathepsin D	Lysosomal autophagy	4.50
**18.**	115718	Cathepsin D		12.33
**19.**	148671816	Carbonyl reductase 3	Antioxidation	2.98
**20.**	148671816	Carbonyl reductase 3		7.80
**A.**	148684825	Protein kinase C, δ binding protein (SRBC/Cavin3)	Caveolae formation	0.24
**B.**	148684825	Protein kinase C, δ binding protein (SRBC/Cavin3)		0.33
**C.**	12846147	Eukaryotic translation initiation factor 2, subunit 1	Translation initiator	0.02
**D.**	109733318	Polymerase I and transcript release factor (PTRF/Cavin1)	Caveolae formation	0.12
**E.**	13431905	γ-synuclein	?	0.32
**F.**	127129	Myosin light chain 1/3, skeletal muscle isoform	Myosin component	0.15
**G.**	109733318	Polymerase I and transcript release factor (PTRF/Cavin1)	Caveolae formation	0.03
**H.**	127129	Myosin light chain 1/3, skeletal muscle isoform	Myosin component	0.16
**I.**	109733318	Polymerase I and transcript release factor (PTRF/Cavin1)	Caveolae formation	0.21
**J.**	109730281	EH-domain containing 2 (EHD2)	Caveolae formation	0.13
**K.**	123247212	Novel protein (2810405K02Rik)	?	0.28
**L.**	116849	Cofilin, non-muscle isoform	Actin depolymerization	0.04

1 HBM, SBO-based high-fat diet containing high dose BMSO; HS, SBO-based high-fat diet without BMSO.

### Validation of Proteomic Data by Western Blotting

**Figure 3:**
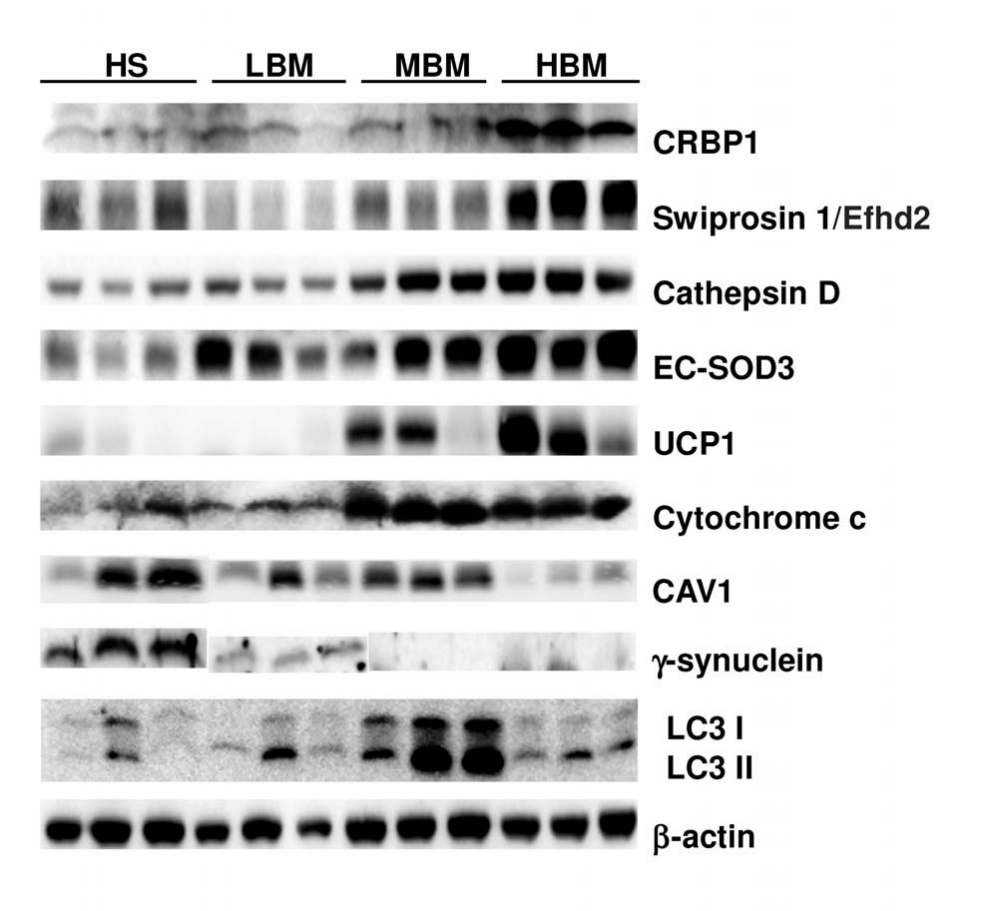
WAT proteins differentially expressed in different groups of BMSO-fed mice validated by immunoblotting. Detection of cellular retinol binding protein 1 (CRBP1), swiprosin 1/Efhd2, cathepsin D, extracellular superoxide dismutase 3 (EC-SOD3), uncoupling protein 1 (UCP1), cytochrome c, caveolin 1 (CAV1), γ-synuclein, and light chain 3 (LC3) in the retroperitoneal fat of mice fed the SBO-based high-fat diet alone (HS) or containing low (LBM), medium (MBM), or high (HBM) percentages of BMSO. β-actin was used as the loading control.

Based on the functions of the 23 identified proteins, including mitochondrial protein, caveolae formation and autophagy (Table 1), the differential expression of 5 from the list and of 4 used as markers for WAT browning (UCP1), mitochondria (cytochrome c), caveolae (CAV1), and autophagy (LC3) was examined by Western blotting of the retroperitoneal fat from the four groups of mice. As shown in Figure 3, BMSO increased levels of CRBP1, swiprosin 1/efhd2, cathepsin D, EC-SOD3, UCP1, and cytochrome c and reduced γ-synuclein and CAV1 levels in the WAT in a dose-dependent manner. Autophagy, as indicated by the shift from LC3 I to LC3 II, was also increased by BMSO.

### Validation of the Presence of “Brite” Adipocytes

We have previously reported that BMSO increases WAT mRNA levels for *Adrb3, Ppara, Ppargc1a,* and *Ucp1* [11], supporting the notion of WAT “browning”. A recent study based on microarray screens of subcloned stroma-vascular cells from inguinal fat identified TMEM26, CD137, and TBX1 as markers for brite adipocytes and EVA1 as a marker for brown adipocytes [16]. In our present study, mRNA levels for brite-, but not brown-, specific genes in the retroperitoneal fat in the HBM group were higher than those in the HS control (Table 2, *P*<0.05 for *Tmem26* and *Tbx1*).

**Table 2:** Levels of mRNAs coding for brite- and brown-specific genes in the retroperitoneal fat of HBM and HS mice 1,2.

mRNA	HS	HBM
	Fold of HS	
Brite-specific gene		
* Tmem26*	1.03±0.11	3.33±0.73*
* CD137*	1.12±0.25	2.20±0.79
* Tbx1*	1.30±0.33	8.16±1.47**
Brown-specific gene		
*Eva1*	1.24±0.75	1.68±1.21

1 Values are the mean ± SE, n = 6.

* *P*<0.05,

** *P*<0.005, different from HS.

2 HBM, SBO-based high-fat diet containing high dose BMSO; HS, SBO-based high-fat diet without BMSO.

### Fatty Acid Composition

The fatty acid composition of total lipids from the epididymal fat of mice indicated that conjugated fatty acids were absent in the HS group (Table S3 **)**. *C*9, *t*11-CLA levels showed a dose-dependent increase in the BMSO-supplemented groups (LBM, MBM and HBM). *C*9, *t*11, *t*13-CLN was only detectable in the HBM group. Similar results were obtained in retroperitoneal fat from another batch of animals (data not shown).

## Discussion

Using proteomics to investigate the global effects of BMSO on the WAT, we showed that WAT levels of 15 proteins [lymphocyte cytosolic protein 1/L-plastin, tropomyosin beta chain, annexin V, swiprosin 1/efhd2, γ-actin, inorganic pyrophosphatase, coactosin-like protein (CLP), CRBP1, mitochondrial ribosomal protein L12 (MRPL12), apolipoprotein A-I preproprotein, demethyl Q7, peroxiredoxin 1, EC-SOD3, cathepsin D, and carbonyl reductase 3] were increased by the HBM diet and levels of protein kinase Cδ binding protein (SRBC/cavin3), eukaryotic translation initiation factor 2, polymerase I and transcript release factor (PTRF/cavin1), γ-synuclein, myosin light chain 1/3, EH-domain containing 2 (EHD2), novel protein, and cofilin were decreased by BMSO. However, our study has certain limitations. First, WAT is a heterogeneous mixture of cells, including stroma-vascular cells, adipocytes, immune cells, and vascular endothelial cells, and the WAT proteome is therefore the average protein abundance in several cell types. Second, our study focused on the pH 4–7 range, so any differentially expressed proteins with pIs outside this range (e.g., UCP1, pI 9.2) would be missed, as would proteins expressed at low levels. Third, since the comparison was made at the end-point of 11 weeks, the sequence of events is unclear. Nevertheless, several trends were apparent and, combined with the histochemical and immunoblotting data, showed that the expression of some novel proteins participating in the processes of delipidation, inflammation, and browning of WAT was altered by BMSO administration (see below).

Lymphocyte cytosolic protein I, also named L-plastin, which is enriched in monocytes/macrophages and T lymphocytes, cross-links F-actin into tight bundles [22]. γ-actin and tropomyosin beta chain in non-muscle cells are implicated in stabilizing cytoskeleton actin filaments [23], in contrast to cofilin-1, which serves as an actin depolymerizing factor [24]. The actin cytoskeleton is required for immune responses, such as phagocytosis, adhesion, and mobility [22]. The BMSO-induced upregulation of L-plastin, γ-actin, and tropomyosin beta chain and the downregulation of cofilin-1 seen in our study suggest stabilization of the actin cytoskeleton, which was putatively related to an increased inflammatory response, as shown by the increased CD68+ macrophage recruitment (Figure 1B). In line with this notion, the F-actin-binding proteins CLP and swiprosin 1 were also upregulated by BMSO. CLP binds to 5-lipoxygenase and translocates it to the nuclear membrane, leading to its activation and increased production of leukotriene A_4_ and other proinflammatory eicosanoids [25]. Swiprosin 1 has been shown to regulate cytokine expression and activate mast cells autocrinely, these effects being dependent on actin polymerization [26]. Another possibility is that the stabilization of the actin cytoskeleton induced by BMSO might be related to inhibition of adipocyte differentiation and hypertrophy. Pharmacological disruption of actin microfilaments increases adipocyte differentiation in 3T3-L1 cells [27] and intracellular TG accumulation in bovine intramuscular preadipocytes [28].

Caveolae are extremely abundant in adipocytes and are proposed to play a role in fatty acid uptake and lipid droplet growth [29]. CAV1 is an integral component of caveolae, and PTRF/cavin1 and SRBC/cavin3 are regarded as CAV1 adaptor proteins that link PKC to regulatory networks involved in controlling caveolae function [30], [31]. Cells lacking PTRF are defective in caveolae and have lower CAV1 levels [30]. Targeted inactivation of either *Cav1* or *Ptrf* results in phenotypes of diet-induced obesity resistance or lipodystrophy in mice [32]–[34]. EH-domains have also been implicated in regulating caveolae-associated endocytosis, vesicle trafficking, and signal transduction [35]. The reduced SRBC/cavin3, PTRF/cavin1, EHD2, and CAV1 protein levels suggest that a reduction in caveolae abundance is involved in the BMSO-mediated delipidative effect on the WAT.

Since MRPL12 is a component of mitochondria ribosomes [36], the increased MRPL12 levels imply a higher content of mitochondria in the WAT in the HBM group compared to the HS control. The increased cytochrome c levels in the BMSO-treated groups support this notion (Figure 3). Recently, a unique function of MRPL12 was revealed, namely that, when not bound to ribosomes, MRPL12, but not other mitochondrial ribosomal proteins, associates with human mitochondria RNA polymerase to activate mitochondria DNA transcription [37]. Demethyl Q7 is another mitochondrial protein shown to be upregulated in the WAT after BMSO administration. Encoded by *Coq7/Clk-1*, demethyl Q7 is required for biosynthesis of coenzyme Q (ubiquinone, CoQ), an essential cofactor in mitochondrial respiration [38], and for the uncoupling function of UCP [39]. Recently, a report showed that, in mice, supplementation with wild bitter melon results in increased mitochondria biogenesis in the skeletal muscle and epididymal fat [40]. This increased mitochondria content, accompanied by the increase in inorganic pyrophosphatase observed in our study, shifts lipid metabolism toward fatty acid β-oxidation, rather than lipogenesis, in BMSO-treated WAT. Thus, taking together the morphological changes and increased mitochondrial protein levels seen in the present study and the previously observed increased mRNA levels for *Adrb3, Ppara, Ppargc1a,* and *Ucp1* [11], these results strongly support the idea that a browning reaction occurs in the WAT of BMSO-fed mice and that this might contribute to the observed resistance to diet-induced obesity and the favorable effects on insulin sensitivity [17]–[19].

In accordance with a study on human BAT (which has been shown to be composed of cells more like murine brite cells, rather than classical brown adipocytes [16], [52]) showing that expression of *Prdm16* or *Pgc1a* is highly correlated with expression of brite-specific, but not brown-specific, genes [41], we showed that brite-specific genes, but not brown-specific genes, were upregulated (Table 2) and that this was accompanied by BMSO-mediated browning. Whether this result is linked to increased differentiation or proliferation of brite precursor cells awaits further study. The coexistence of inflammation and browning in the WAT is not unexpected, since induction of COX2, a downstream effector of β-adrenergic signaling, has been demonstrated to be required for the induction of brown fat-like cells in WAT depots [42]. In addition, alternatively activated macrophages in the WAT can serve as a source of adrenaline and noradrenaline, in addition to sympathetic nerves [43].

The upregulation of cathepsin D by BMSO seen in our study may indicate that active lysosomal autophagy occurs in the cells of the WAT that are undergoing dynamic changes and remodeling. We previously demonstrated that BMSO increases WAT apoptosis, shown using the TUNEL assay [11]. In the present study, the changes in LC3 protein expression also showed that BMSO increased autophagy (Figure 3). Increased autophagy has been seen in CAV1-deficient adipocytes [44]. On the other hand, cathepsin D triggers apoptosis in response to ROS-induced permeabilization of lysosomes [45]. The increased levels of EC-SOD3, carbonyl reductase 3, and peroxiredoxin 1 might reflect increased ROS production in the WAT in BMSO-fed mice. In line with this, the thiobarbituric acid reactive substances (TBARS)/TG ratio in the WAT was significantly increased by BMSO administration (HS vs. HBM, 1.63±1.41 vs. 5.74±1.86 µmol/mg). TBARS levels were used as a measure of lipid peroxidation products and this value was corrected for TG levels, since there were large differences in TG content in the WAT in the HS and HBM groups. The oxidative stress might be ascribed to CLN, which is abundant in BMSO and easily oxidized, or to ROS production during inflammation and apoptosis.

In the WAT, CRBP1 expression is restricted to preadipocytes, and CRBP1, like pref-1, is regarded as a preadipocyte marker [46]. Since CRBP1 is undetectable in endothelial cells, macrophages, or mature adipocytes [47], the increased CRBP1 levels seen in our study reveal an increased population of preadipocytes in the WAT of BMSO-fed mice and imply downregulation of PPARγ activity.

In the present study, BMSO induced WAT delipidation, apoptosis, inflammation, and browning, reminiscent of the situation in the WAT of CLA-fed mice [2], [3], [48], [49]. Since *c*9,*t*11-CLA has no anti-adiposity function [2], [3], the effects of BMSO observed in this study cannot be attributed to CLA derived from CLN, since CLN is reported to be metabolized into *c*9,*t*11-CLA, rather than *t*10,*c*12-CLA [50], and since the fatty acid composition of the WAT in our study confirmed that *c*9,*t*11-CLA, but not *t*10,*c*12-CLA, was detectable in the BMSO-supplemented groups (Table S3). A recent study showed that, when mice were fed with CLA at a much lower dose (0.1% *t*10,*c*12-CLA or 0.2% CLA mixture) without causing lipodystrophy or hepatic steatosis, marked browning coupled with inflammation in WAT was observed [51]. We speculate that CLN and CLA might share a common metabolic or signaling pathway, leading to a qualitatively similar outcome in the WAT.

**Figure 4:**
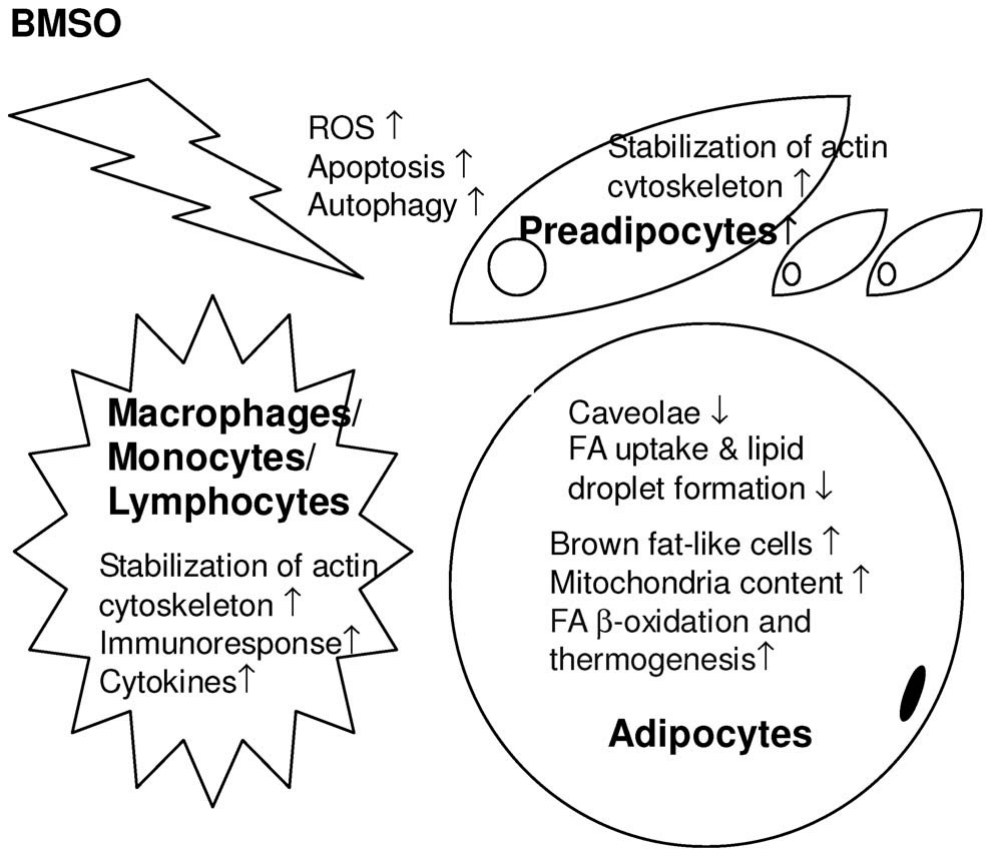
The proteomic results combined with the histological and immunoblotting data reveal global changes in the WAT caused by long-term high dose BMSO administration. The BMSO-mediated delipidative, inflammatory, and browning effects in the WAT are associated with reduced caveolae formation, increased ROS levels, tissue remodeling/repair (apoptosis and autophagy), brown fat-like cell recruitment, and actin cytoskeleton stabilization. The diagram shows a hypothetical scheme, as it is not known in what cells these changes occur.

In summary, the proteomic analysis combined with the immunoblotting and histological data showed that the WAT in mice fed chronic high dose BMSO showed reduced caveolae formation, increased ROS insult, tissue remodeling/repair, mitochondria uncoupling, actin cytoskeleton stabilization, and an increased inflammation response, as shown in Figure 4. Considering that adult human BAT bears the molecular characteristics of murine brite cells [16], [52], manipulation of adipocyte differentiation in order to promote more brown-like adipocytes within the WAT, i.e. browning or beigeing, has been suggested as a novel approach to the treatment of obesity and its associated problems [17]–[19]. Dietary supplementation with BMSO or conjugated fatty acids to increase WAT browning therefore holds great promise for the treatment of obesity and metabolic syndrome.

## Supporting Information

Figure S1 **Amplification of spots derived from a protein that is differentially expressed in the HS and HBM groups.**
(DOCX)

Table S1 **Sequences of the PCR primers.**
(DOCX)

Table S2 **The theoretical and experimental isoelectric point and molecular weight of proteins identified.**
(DOCX)

Table S3 **Fatty acid composition of lipids extracted from the epididymal fat of the mice fed a SBO-based high-fat diet containing different doses of BMSO for 11 wk.**
(DOCX)
