# Supplementary material for: Altered White Adipose Tissue Protein Profile in C57BL/6J Mice Displaying Delipidative, Inflammatory, and Browning Characteristics after Bitter Melon Seed Oil Treatment
Source: PLoS One. 2013 Sep 6;8(9):e72917. doi: 10.1371/journal.pone.0072917 (PMC3765199; doi:10.1371/journal.pone.0072917)
Supplement: Figure S1 — Amplification of spots derived from a protein that is differentially expressed in the HS and HBM groups. (DOCX) [file pone.0072917.s001.docx]

**Figure S1. Amplification of spots derived from a protein that is differentially expressed in the HS and HBM groups.**
