# Supplementary material for: Altered White Adipose Tissue Protein Profile in C57BL/6J Mice Displaying Delipidative, Inflammatory, and Browning Characteristics after Bitter Melon Seed Oil Treatment
Source: PLoS One. 2013 Sep 6;8(9):e72917. doi: 10.1371/journal.pone.0072917 (PMC3765199; doi:10.1371/journal.pone.0072917)
Supplement: Table S1 — Sequences of the PCR primers. (DOCX) [file pone.0072917.s002.docx]

**Table S1.** Sequences of the PCR primers

| **Gene (Accession number)** | **Primer** |
| --- | --- |
| *Tmem26* (NM_177794.3) | ACCCTGTCATCCCACAGAG (F)  TGTTTGGTGGAGTCCTAAGGTC (R) |
| *CD137* (NM_001077509.1) | CGTGCAGAACTCCTGTGATAAC(F) GTCCACCTATGCTGGAGAAGG (R) |
| *Tbx1* (NM_011532.1) | GGCAGGCAGACGAATGTTC(F)  TTGTCATCTACGGGCACAAAG (R) |
| *Eva1* (NM_007962.4) | CCACTTCTCCTGAGTTTACAGC (F)  GCATTTTAACCGAACATCTGTCC (R) |
