# Supplementary material for: Altered White Adipose Tissue Protein Profile in C57BL/6J Mice Displaying Delipidative, Inflammatory, and Browning Characteristics after Bitter Melon Seed Oil Treatment
Source: PLoS One. 2013 Sep 6;8(9):e72917. doi: 10.1371/journal.pone.0072917 (PMC3765199; doi:10.1371/journal.pone.0072917)
Supplement: Table S2 — The theoretical and experimental isoelectric point and molecular weight of proteins identified. (DOCX) [file pone.0072917.s003.docx]

**Table S2.** The theoretical and experimental isoelectric point and molecular weight of proteins identified **^1^**

| **SN** | **T (kD/pI)** | **E (kD/pI)** | **MP** | **C (%)** |
| --- | --- | --- | --- | --- |
| 1. | 70.2/5.2 | 73.9/5.2 | 29 | 31 |
| 2. | 70.2/5.2 | 73.5/5.3 | 54 | 42 |
| 3. | -- | 39.4/4.5 | -- | -- |
| 4. | 32.8/4.7 | 40.0/4.6 | 106 | 91 |
| 5. | 35.8/4.8 | 33.6/4.8 | 5 | 14 |
| 6. | 26.8/5.1 | 32.9/5.0 | 25 | 45 |
| 7. | 41.8/5.3 | 41.3/5.1 | 26 | 42 |
| 8. | 32.7/5.4 | 35.5/5.3 | 9 | 26 |
| 9. | 15.9/5.2 | 16.7/4.9 | 15 | 44 |
| 10. | 15.9/5.1 | 16.2/5.3 | 12 | 65 |
| 11. | -- | 16.3/6.0 | -- | -- |
| 12. | 21.7/9.3 | 21.9/5.8 | 12 | 29 |
| 13. | 30.6/5.5 | 25.9/5.6 | 37 | 61 |
| 14. | 24.0/6.7 | 21.0/6.5 | 12 | 27 |
| 15. | 18.9/6.8 | 24.8/6.6 | 19 | 45 |
| 16. | 27.4/6.2 | 30.3/6.4 | 4 | 20 |
| 17. | 45.0/6.7 | 31.0/6.6 | 11 | 16 |
| 18. | 45.0/6.7 | 30.4/6.6 | 9 | 19 |
| 19. | 31.0/6.2 | 32.1/6.7 | 24 | 60 |
| 20. | 31.0/6.2 | 32.0/6.8 | 4 | 14 |
| A. | 27.9/5.5 | 37.7/5.8 | 49 | 55 |
| B. | 27.9/5.5 | 37.7/5.6 | 39 | 50 |
| C. | 36.1/5.0 | 37.2/5.3 | 7 | 19 |
| D. | 44.0/5.4 | 40.5/5.7 | 6 | 14 |
| E. | 13.2/4.7 | 18.7/4.6 | 48 | 94 |
| F. | 20.6/5.0 | 24.1/5.0 | 13 | 35 |
| G. | 44.0/5.4 | 23.7/5.0 | 8 | 17 |
| H. | 20.6/5.0 | 23.9/5.4 | 6 | 19 |
| I. | 44.0/5.4 | 23.6/5.4 | 20 | 24 |
| J. | 61.2/6.1 | 33.7/6.4 | 9 | 15 |
| K. | 21.7/6.3 | 24.6/6.7 | 21 | 47 |
| L. | 18.6/8.2 | 20.6/6.7 | 4 | 22 |

^1^ C, protein sequence coverage in percentage; E, experimental values; MP, number of matched peptides; SN, spot number; T, theoretical values.
