# Supplementary material for: Altered White Adipose Tissue Protein Profile in C57BL/6J Mice Displaying Delipidative, Inflammatory, and Browning Characteristics after Bitter Melon Seed Oil Treatment
Source: PLoS One. 2013 Sep 6;8(9):e72917. doi: 10.1371/journal.pone.0072917 (PMC3765199; doi:10.1371/journal.pone.0072917)
Supplement: Table S3 — Fatty acid composition of lipids extracted from the epididymal fat of the mice fed a SBO-based high-fat diet containing different doses of BMSO for 11 wk. (DOCX) [file pone.0072917.s004.docx]

**Table S3.** Fatty acid composition of lipids extracted from the epididymal fat of the mice fed a SBO-based high-fat diet containing different doses of BMSO for 11wk ^1,2,3,4^

| **Fatty acid** | **HS** | **LBM** | **MBM** | **HBM** |
| --- | --- | --- | --- | --- |
| *Saturated fatty acids* | % total fatty acids | | | |
| C12:0 | 0.02±0.00^b^ | 0.03±0.00^b^ | 0.05±0.00^a^ | 0.06±0.01^a^ |
| C14:0 | 0.42±0.03^b^ | 0.53±0.01^ab^ | 0.61±0.02^ab^ | 0.76±0.14^a^ |
| C16:0 | 14.20±0.60 | 13.33±0.11 | 14.96±0.51 | 17.75±3.03 |
| C18:0 | 2.77±0.05^b^ | 3.06±0.14^b^ | 4.29±0.15^ab^ | 7.96±2.06^a^ |
| C20:0 | 0.08±0.01 | 0.16±0.06 | 0.09±0.01 | 0.22±0.09 |
| *Monounsaturated fatty acids* |  |  |  |  |
| C14:1 | ND^b^ | 0.03±0.00^a^ | 0.04±0.00^a^ | 0.03±0.01^a^ |
| C16:1, n-7 | 0.45±0.02^c^ | 0.56±0.04^bc^ | 0.73±0.03^b^ | 1.14±0.10^a^ |
| C16:1, n-9 | 1.84±0.20^b^ | 3.31±0.30^a^ | 3.95±0.17^a^ | 3.98±0.11^a^ |
| C18:1, n-9 | 30.27±0.73^c^ | 32.44±0.90^bc^ | 37.37±0.30^b^ | 44.43±2.92^a^ |
| C20:1, n-9 | 0.13±0.12 | ND | ND | 0.40±0.19 |
| *Polyunsaturated fatty acids* |  |  |  |  |
| C18:2, n-6（LA） | 46.09±1.44^a^ | 39.08±0.80^ab^ | 30.71±0.65^b^ | 17.19±6.60^c^ |
| C18:3, n-3（LN） | 3.31±0.31^a^ | 2.81±0.16^a^ | 1.66±0.04^b^ | 0.70±0.36^c^ |
| *c*9, *t*11-CLA | ND^b^ | 3.03±1.32^ab^ | 5.34±0.27^ab^ | 6.39±0.48^a^ |
| *t*10, *c*12-CLA | ND | ND | ND | ND |
| *c*9, *t*11, *t*13-CLN | ND | ND | ND | 0.08±0.07 |
| C20:2, n-6 | 0.16±0.01 | 1.39±1.08 | 0.10±0.01 | ND |
| C20:3, n-6 | 0.17±0.08^a^ | 0.20±0.02^a^ | ND^b^ | ND^b^ |
| C20:4, n-6 | ND^b^ | 0.02±0.15^b^ | 0.11±0.01^ab^ | 0.22±0.07^a^ |
| C22:5, n-6 | ND | ND | ND | 0.61±0.50 |
| C20:5, n-3 | ND^b^ | 0.02±0.02^b^ | 0.11±0.01^b^ | 0.26±0.08^a^ |
| C22:5, n-3 | 0.08±0.00 | ND | ND | 0.12±0.10 |

^1^ The values are the mean ± S.E. (n=4).

^2^ The significance of differences among the four groups was analyzed statistically by one-way ANOVA and Duncan’s multiple range test. Values not sharing the same superscript letter in the same row are significantly different among groups (*P*＜0.05).

^3^ ND: not detectable, <0.01%.

^4^ HBM, SBO-based high-fat diet containing high dose BMSO; HS, SBO-based high-fat diet; LBM, SBO-based high-fat diet containing low dose BMSO; MBM, SBO-based high-fat diet containing medium dose BMSO.
